# The correlation between epithelial–mesenchymal transition classification and MMP2 expression of circulating tumor cells and prognosis of advanced or metastatic nasopharyngeal carcinoma

**DOI:** 10.1515/med-2024-1074

**Published:** 2025-03-11

**Authors:** Xiaoju Wang, Yuxin Zhang, Yiqing Wang, Lei Shi, Caiqin Yuan, Wei Yin, Yaoshu Teng, Jing Li, Yanjiao Mao

**Affiliations:** Radiotherapy Department, Hangzhou Cancer Hospital, Hangzhou, 310005, Zhejiang, China; ENT Department, Hangzhou First People’s Hospital, Hangzhou, 310005, Zhejiang, China

**Keywords:** epithelial–mesenchymal transition, circulating tumor cells, nasopharyngeal carcinoma, MMP2, prognostic value

## Abstract

**Background:**

Epithelial–mesenchymal transition (EMT) and circulating tumor cells (CTCs) are key prognostic factors in nasopharyngeal carcinoma (NPC). However, the role of EMT status in CTCs for predicting outcomes in advanced NPC treated with radiotherapy after induction chemotherapy remains unclear.

**Methods:**

A total of 143 CTC tests from 95 advanced/metastatic NPC patients were analyzed before, during, and after radiotherapy, with a 60-month follow-up. CTC count, matrix metalloproteinase 2 (MMP2)) protein expression, and EMT subtypes were examined.

**Results:**

During radiotherapy, CTC counts increase but decrease afterward. Patients with higher pre-radiotherapy tumor-node-metastasis (TNM) stages have lower total and M-subtype CTC counts. Higher T and TNM stages during radiotherapy correlate with increased EMT-state CTCs, especially hybrid CTCs. EA/IgG-positive patients have a higher number of hybrid CTCs and E-type (epithelial + hybrid) CTCs, while EBV-EA-negative patients have more mesenchymal CTCs. A higher post-radiotherapy CTC count predicts relapse, and the positive rate of MMP2 expression on hybrid and epithelial CTCs is higher than that on mesenchymal CTCs.

**Conclusion:**

EMT status, particularly in hybrid CTCs, is a potential prognostic marker for relapse in advanced NPC after radiotherapy.

## 1 Introduction

Nasopharyngeal carcinoma (NPC) is a malignant tumor that occurs in the nasopharyngeal area of the upper respiratory tract [1]. Due to its unique geographical distribution, distinct pathological types, and close association with the Epstein-Barr Virus (EBV), NPC has become a unique and important field in the study of head and neck tumors [2,3]. NPC in a stable, non-progressive stage is typically manageable, largely owing to its positive response to both radiotherapy and chemotherapy [4]. On the other hand, NPC that is progressing tends to aggressively metastasize to cervical lymph nodes and other organs. The presence of metastatic disease is a marker of treatment failure and is associated with a poor prognosis in NPC patients [5,6]. Distant metastasis is the leading cause of cancer-related death in patients with NPC, and the median overall survival (OS) of NPC patients with recurrent or primary metastatic disease is 20 months [7].

Circulating tumor cells (CTCs) are often regarded as the precursors of metastasis, originating from the release of cancer cells into the bloodstream from primary and secondary tumors [8,9]. Identifying these scarce CTCs in the bloodstream opens up promising prospects for the early and sensitive detection of metastatic cancer cells. The effectiveness of CTCs in assessing treatment response and predicting outcomes has been established in a range of metastatic cancers [10,11]. The process of enhancing and isolating these infrequent CTCs in the blood has seen significant advancements, paving the way for the innovative use of blood-based cancer diagnostics to actively monitor the progression of cancer and the response to treatment in real-time. Ou and his colleagues reported that CTCs could be an independent prognostic value for advanced NPC [12]. CTCs exhibit a high degree of heterogeneity, particularly as they undergo the epithelial–mesenchymal transition (EMT) process [13,14]. During EMT, tumor cells undergo a transformation where they shed their epithelial attributes, such as robust adhesion to neighboring cells and the basal membrane. Concurrently, they acquire more aggressive traits, including enhanced motility, the capacity to break down the extracellular matrix (ECM), and characteristics akin to stem cells [15].

The ECM plays a crucial role in providing biochemical support to adjacent cells and modulating the microenvironmental changes that occur during tumor development [16]. Matrix metalloproteinases (MMPs), also known as matrixins, comprise a group of endopeptidases that are dependent on calcium and contain zinc. These enzymes are key in the remodeling of the ECM, as they regulate the breakdown of ECM components, including the connective tissue matrices [17]. Within the MMP family, MMP2 is recognized as a crucial contributor to the progression of metastasis including NPC. For example, MMP2 has been proven to play a functional role in promoting cell migration and invasion in NPC through Capn4 mediation [18].

Although the prognostic role of CTCs in NPC has been clarified, the relationship between different EMT states of CTCs, their expression of MMP2, and the prognosis of patients with advanced NPC has not yet been thoroughly investigated. In this study, we explored the relationship between different EMT states of CTCs and the prognosis of patients with advanced NPC undergoing radiotherapy, and further investigated the gene expression distribution of *MMP2* in CTCs across different EMT states.

## 2 Methods

### 2.1 Patient enrollment

This study included 95 patients with advanced progressive NPC treated at the Hangzhou Cancer Hospital. The patient recruitment criteria were as follows: (1) aged between 18 and 80 years, and had not received intravenous chemotherapy at the time of enrollment; (2) expected survival time of at least 3 months; (3) normal liver and kidney function; (4) no planned surgeries within 6 months after the start of the trial; and (5) other organ tumors excluded by imaging findings. Clinical and laboratory characteristics of all the patients, including original lesion diameter, status of lymph node metastasis, tumor-node-metastasis (TNM) stage, EBV serology, cancer recurrence status, etc., were collected.

### 2.2 Isolation and detection of CTCs

CTC analysis was performed using the CanPatrol CTC enrichment system and *in situ* hybridization (ISH) technology before, during, and after chemotherapy. Peripheral blood samples were collected (5 mL, treated with ethylenediaminetetraacetic acid as an anticoagulant) after the initial 2 mL of blood was discarded. A lysis buffer specifically designed for red blood cells was employed to eliminate these cells, followed by a resuspension in phosphate-buffered saline containing 4% formaldehyde (Sigma, St Louis, MO, USA) for a duration of 5 min prior to the filtration process. The isolation of CTCs was accomplished through the use of the CanPatrol CTC enrichment system, which incorporates a filtration tube equipped with a membrane featuring pores of 8 μm in diameter (Sur Exam, Guangzhou, China), along with a vacuum plate manifold that includes a valve adjustment (SurExam, Guangzhou, China), an E-Z96 vacuum manifold (Omega, Norcross, GA, USA), and a vacuum pump (Auto Science, Tianjin, China). The RNA ISH method was utilized to detect and analyze the expression levels of epithelial and mesenchymal genes within the CTCs, employing three different types of nucleic acid probes. The targets for detection encompassed *CD45*, *EPCAM*, *CK8/18/19* (markers for epithelial cells), *VIMENTIN*, and *TWIST* (markers for mesenchymal cells). The sequences used for RNA ISH are as follows:

*EPCAM*: TGGTGCTCGTTGATGAGTCAAGCCAGCTTTGAGCAAATGAAAAGCCCATCATTGTTCTGGCTCTCATCGCAGTCAGGATCTCCTTGTCTGTTCTTCTGACCTCAGAGCAGGTTATTTCAG;

*CK8*: CGTACCTTGTCTATGAAGGAACTTGGTCTCCAGCATCTTGCCTAAGGTTGTTGATGTAGCCTGAGGAAGTTGATCTCGTCCAGATGTGTCCGAGATCTGGTGACCTCAGCAATGATGCTG;

*CK18*: AGAAAGGACAGGACTCAGGCGAGTGGTGAAGCTCATGCTGTCAGGTCCTCGATGATCTTGCAATCTGCAGAACGATGCGGAAGTCATCAGCAGCAAGACGCTGCAGTCGTGTGATATTGG;

*CK19*: CTGTAGGAAGTCATGGCGAGAAGTCATCTGCAGCCAGACGCTGTTCCGTCTCAAACTTGGTTCTTCTTCAGGTAGGCCAGCTCAGCGTACTGATTTCCTCGTGAACCAGGCTTCAGCATC;

*VIMENTIN*: GAGCGAGAGTGGCAGAGGACCTTTGTCGTTGGTTAGCTGGCATATTGCTGACGTACGTCAGAGCGCCCCTAAGTTTTTAAAAGATTGCAGGGTGTTTTCGGGCCAATAGTGTCTTGGTAG;

*TWIST*: ACAATGACATCTAGGTCTCCCTGGTAGAGGAAGTCGATGTCAACTGTTCAGACTTCTATCCCTCTTGAGAATGCATGCATTTTCAGTGGGCTGATTGGCACTTACCATGGGTCCTCAATAA;

*CD45*: TCGCAATTCTTATGCGACTCTGTCATGGAGACAGTCATGTGTATTTCCAGCTTCAACTTCCCATCAATATAGCTGGCATTTTGTGCAGCAATGTATTTCCTACTTGAACCATCAGGCATC.

*MMP2: CAACTCTTTGTCCGTTTTGGAAGGTGTTCAGGTATTGCACCAAACAGGTTGCAGCTCTCCTTCTTTAGTGTGTCCTTCAGCAGTCCAAAGAACTTCTGCAGGTCAAGATCACCTGTCTGGCGCATGGTCTCGATGGTATTGTTGTAGGCCACATCTG*

Based on the gene expression levels observed, CTCs were classified into mesenchymal CTCs, epithelial CTCs, and hybrid CTCs, with the latter exhibiting fluorescence indicative of both epithelial and mesenchymal gene expressions. Moreover, to evaluate the expression of MMP2 in CTCs, we utilized immunohistochemistry for detection (Invitrogen, 1:1,000, Catalog # 35-1300Z).

### 2.3 Statistical analysis

Chi-square tests or Spearman correlation were used to analyze the association between CTCs and clinical pathological characteristics. Univariate variance analysis was employed to compare CTC counts at different time points of radiotherapy in NPC patients. A *P*-value of <0.05 was considered statistically significant. All statistical analyses were performed using SPSS software version 25.0.


**Consent to participate:** Written informed consent was obtained from individual or guardian participants.
**Ethical approval:** The experimental protocol was established, according to the ethical guidelines of the Helsinki Declaration and was approved by the Human Ethics Committee of the Hangzhou Cancer Hospital (Ethics Approval Number: HZCH-2022).

## 3 Results

### 3.1 Differences in CTC detection during different radiotherapy treatment periods

We analyzed the changes in CTC counts and their subtypes during different phases of radiation therapy. The results showed that the detection rates of total CTCs before, during, and after radiation therapy were 91.7, 90.1, and 78.9%, respectively, with median total CTC counts of 4, 7, and 2. Compared to before radiation therapy, there was an increase in total CTC counts during treatment, followed by a decrease after the treatment (Table 1). Interestingly, mesenchymal-type CTCs showed the same trend in positivity rate and total CTC count, with rates before, during, and after radiation therapy of 54.2, 59.1, and 49.1%, respectively. However, there was no significant difference in the median values among the three time points in the statistical analysis (Table 1).

**Table 1:** Overview of CTC detection data at different clinical stages

Clinical stages	Total number of CTCs			Mesenchymal CTCs		
	Positivity rate (%)	Median value	Total range	Positivity rate (%)	Median value	Quantity range
After induction chemotherapy, before radiotherapy (*n* = 24)	91.7	4	0–36	54.2	1	0–4
During radiotherapy (*n* = 44)	90.1	7	0–40	59.1	1	0–18
First time after radiotherapy (*n* = 57)	78.9	2	0–40	49.1	0	0–9

### 3.2 Correlation between tumor clinical features and the number and subtypes of CTCs detected at different times during radiotherapy

**Figure 1:**
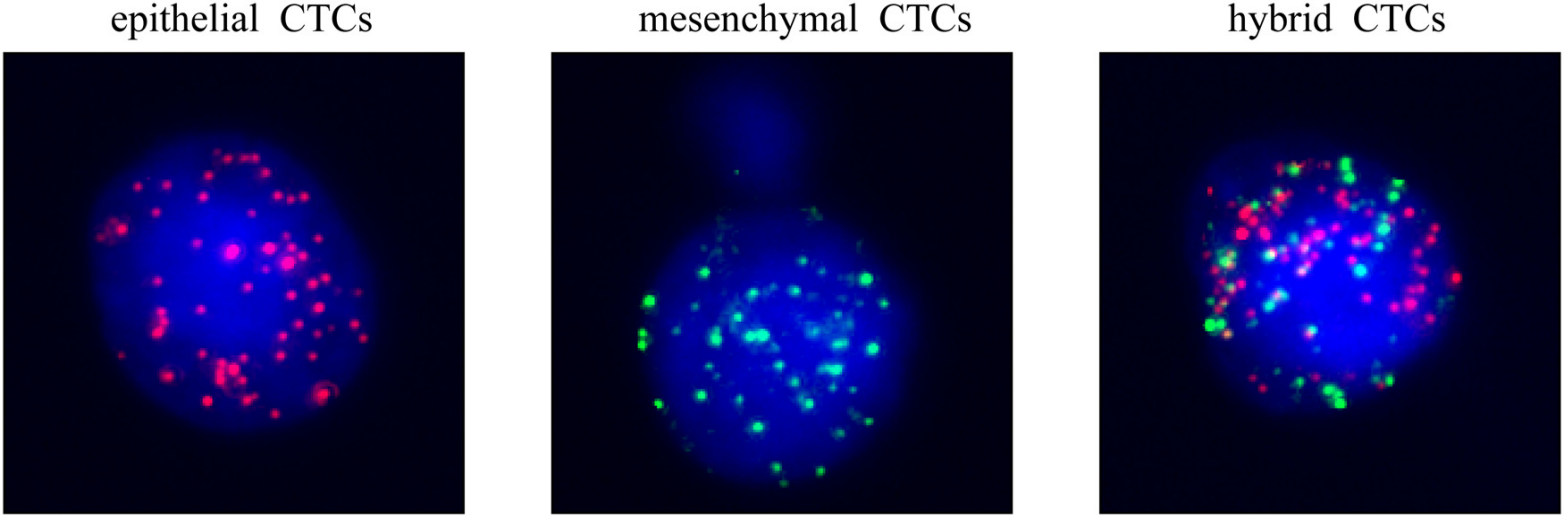
Representative images of different types of CTCs. The epithelial CTCs had red fluorescence, the hybrid CTCs had both red and green fluorescence, and the mesenchymal CTCs had green fluorescence.

We classified CTCs into different EMT statuses based on the expression of epithelial and mesenchymal genes on the CTCs (Figure 1). To further analyze the relationship between CTCs and patient clinical characteristics, we conducted a correlation analysis of the total CTC count and CTC counts of different EMT statuses at various stages of radiotherapy with patient clinical features. These clinical features include tumor size, tumor T stage, the presence of lymph node metastasis, TNM staging, EBV capsid antigen (EBV-CA), EBV early antigen (EBV-EA), EBV-DNA, and whether NPC has recurred or progressed. Before radiotherapy in NPC patients, only the total count of CTCs was negatively correlated with the TNM staging of the patients, meaning that patients with a higher TNM stage had a lower total count of CTCs. No correlation was found between other indicators (Table 2). During radiotherapy in patients with NPC, the number and types of CTCs – including hybrid CTCs, epithelial-type CTCs (epithelial + hybrid), and mesenchymal-type CTCs (mesenchymal + hybrid)  – were positively correlated with the pre-treatment T stage and TNM stage. This means that patients with higher T stages and TNM stages had a greater number or individual types of CTCs; whereas, the count of mesenchymal-type CTCs was only positively correlated with the pre-treatment T stage (Table 3). The relationship between CTCs and EBV markers shows that EA/IgG is positively correlated with the number of hybrid CTCs and epithelial-type (epithelial + hybrid) CTCs at this time. Patients positive for EA/IgG have higher counts of these two types of CTCs; meanwhile, patients who are negative for EBV-EA are more likely to have a positive detection of mesenchymal-type CTCs (Table 3). After radiotherapy in patients with NPC, the total number and types of CTCs – including hybrid CTCs, epithelial-type CTCs (epithelial + hybrid), and mesenchymal-type CTCs (mesenchymal + hybrid) – are negatively correlated with progression, meaning that patients with higher counts or types of CTCs are more likely to experience a recurrence (Table 4).

**Table 2:** Relationship between CTCs and different clinical characteristics after induction chemotherapy and before radiotherapy

Spearman Rho			Total number of CTCs	Number of epithelial CTCs		Number of hybrid CTCs	Number of mesenchymal CTCs	Total number of CTCs (<5/≥5)	Number of epithelial-type CTCs	Number of mesenchymal -type CTCs	Mesenchymal CTCs (negative/positive)
Tumor size	Correlation coefficient	*N* = 19	0.103	−0.155		0.196	0.000	0.080	−0.031	0.062	0.164
	*P* value		0.673	0.527		0.421	1.000	0.746	0.901	0.800	0.503
T stage (1–2/3–4)	Correlation coefficient	*N* = 23	0.027	−0.014		−0.035	−0.171	0.095	0.027	0.014	0.016
	*P* value		0.902	0.950		0.873	0.435	0.666	0.903	0.951	0.944
Lymph node metastasis (N0/N+)	Correlation coefficient	*N* = 23	0.212	−0.048		0.147	−0.066	0.247	0.094	0.082	0.041
	*P* value		0.332	0.830		0.504	0.765	0.255	0.671	0.710	0.854
TNM stage (1–2/3/4)	Correlation coefficient	*N* = 24	−0.408	−0.395		−0.243	−0.389	−0.422	−0.282	−0.452	−0.324
	*P* value		0.048	0.056		0.252	0.060	0.040	0.182	0.027	0.123
EBV-CA (negative/positive)	Correlation coefficient	*N* = 21	0.000	−0.132		−0.059	−0.181	0.032	−0.084	−0.093	−0.085
	*P* value		1.000	0.568		0.800	0.431	0.890	0.718	0.688	0.713
EBV-EA (negative/positive)	Correlation coefficient	*N* = 21	0.228	0.091		−0.147	0.080	0.085	0.269	0.033	−0.159
	*P* value		0.320	0.695		0.526	0.732	0.714	0.238	0.888	0.491
EA/IgG (negative/positive)	Correlation coefficient	*N* = 7	0.612	0.412		−0.428	NA	0.471	0.618	0.408	−0.471
	*P* value		0.144	0.358		0.338	NA	0.286	0.139	0.363	0.286
EBV-DNA (negative/positive)	Correlation coefficient	*N* = 17	0.113	−0.013		0.200	0.112	−0.044	0.000	0.152	0.044
	*P* value		0.665	0.961		0.441	0.668	0.868	1.000	0.560	0.868
Disease recurrence/progression (yes/no)	Correlation coefficient	*N* = 19	0.010	0.184		0.259	0.094	0.131	0.060	0.151	0.288
	*P* value		0.967	0.451		0.283	0.703	0.593	0.806	0.538	0.233

**Table 3:** Relationship between CTCs and different clinical characteristics during radiotherapy

Spearman Rho			Total number of CTCs	Number of epithelial CTCs	Number of hybrid CTCs	Number of mesenchymal CTCs	Total number of CTCs (<5/≥5)	Number of epithelial-type CTCs	Number of mesenchymal -type CTCs	Mesenchymal CTCs (negative/positive)
Tumor size	Correlation coefficient	*N* = 41	0.153	−0.139	0.191	−0.017	0.033	0.155	0.187	−0.082
	*P* value		0.338	0.387	0.230	0.915	0.837	0.334	0.242	0.612
T stage (1–2/3–4)	Correlation coefficient	*N* = 42	0.303	−0.060	0.340	−0.023	0.303	0.324	0.310	−0.096
	*P* value		0.051	0.707	0.028	0.887	0.051	0.036	0.046	0.544
Lymph node metastasis (N0/N+)	Correlation coefficient	*N* = 42	−0.004	−0.099	−0.004	0.080	0.000	0.008	−0.011	0.133
	*P* value		0.981	0.531	0.981	0.616	1.000	0.962	0.942	0.400
TNM stage (1−2/3/4)	Correlation coefficient	*N* = 42	0.410	−0.232	0.449	0.335	0.304	0.346	0.490	0.300
	*P* value		0.007	0.139	0.003	0.030	0.050	0.025	0.001	0.054
EBV-CA (negative/positive)	Correlation coefficient	*N* = 40	0.079	−0.144	0.093	−0.232	−0.191	0.090	0.057	−0.303
	*P* value		0.629	0.375	0.570	0.150	0.238	0.582	0.726	0.057
EBV-EA (negative/positive)	Correlation coefficient	*N* = 40	−0.002	−0.123	−0.012	−0.247	−0.310	0.000	−0.028	−0.342
	*P* value		0.988	0.449	0.942	0.125	0.052	1.000	0.862	0.031
EA/IgG (negative/positive)	Correlation coefficient	*N* = 22	0.315	0.187	0.545	0.066	0.239	0.438	0.396	0.174
	*P* value		0.154	0.404	0.009	0.770	0.284	0.041	0.068	0.440
EBV-DNA (negative/positive)	Correlation coefficient	*N* = 37	−0.137	−0.037	−0.146	0.039	0.137	−0.143	−0.106	0.083
	*P* value		0.418	0.826	0.388	0.820	0.420	0.398	0.533	0.625
Disease recurrence/progression (yes/no)	Correlation coefficient	*N* = 40	−0.214	0.028	−0.298	−0.094	−0.053	−0.268	−0.257	−0.118
	*P* value		0.186	0.865	0.062	0.563	0.744	0.094	0.109	0.468

**Table 4:** Relationship between CTCs and different clinical characteristics first time after radiotherapy

Spearman Rho			Total number of CTCs	Number of epithelial CTCs	Number of hybrid CTCs	Number of mesenchymal CTCs	Total number of CTCs (<5/≥5)	Number of epithelial-type CTCs	Number of mesenchymal -type CTCs	Mesenchymal CTCs (negative/positive)
Tumor size	Correlation coefficient	*N* = 43	−0.012	−0.049	−0.048	−0.076	0.016	−0.024	−0.035	−0.137
	*P* value		0.941	0.757	0.759	0.630	0.919	0.876	0.824	0.382
T stage (1–2/3–4)	Correlation coefficient	*N* = 54	−0.050	−0.017	−0.019	−0.047	−0.045	−0.022	−0.055	−0.116
	*P* value		0.722	0.903	0.894	0.738	0.749	0.874	0.695	0.403
Lymph node metastasis (N0/N+)	Correlation coefficient	*N* = 55	0.022	−0.035	−0.008	0.034	−0.049	0.010	−0.016	0.058
	*P* value		0.873	0.801	0.952	0.803	0.722	0.941	0.907	0.677
TNM stage (1–2/3/4)	Correlation coefficient	*N* = 55	0.192	0.222	0.208	0.061	0.064	0.286	0.124	0.193
	*P* value		0.161	0.104	0.127	0.656	0.641	0.034	0.365	0.159
EBV-CA (negative/positive)	Correlation coefficient	*N* = 41	0.018	0.433	0.075	−0.056	0.040	0.154	−0.040	0.012
	*P* value		0.909	0.005	0.639	0.730	0.806	0.338	0.806	0.941
EBV-EA (negative/positive)	Correlation coefficient	*N* = 41	−0.028	0.153	0.090	−0.041	−0.037	0.045	−0.013	0.020
	*P* value		0.863	0.341	0.577	0.801	0.818	0.779	0.936	0.904
EA/IgG (negative/positive)	Correlation coefficient	*N* = 17	0.086	−0.100	0.165	0.051	0.070	0.062	0.099	0.029
	*P* value		0.743	0.702	0.528	0.847	0.788	0.813	0.706	0.913
EBV-DNA (negative/positive)	Correlation coefficient	*N* = 36	0.210	0.123	0.000	0.281	0.235	0.054	0.179	0.278
	*P* value		0.218	0.474	1.000	0.097	0.167	0.752	0.295	0.100
Disease recurrence/progression (yes/no)	Correlation coefficient	*N* = 27	−0.399	−0.117	0.433	−0.170	−0.317	−0.430	−0.401	−0.227
	*P* value		0.039	0.562	0.024	0.396	0.107	0.025	0.038	0.256

### 3.3 Relationship between MMP2 protein expression on CTC and various clinical indicators of NPC

**Figure 2:**
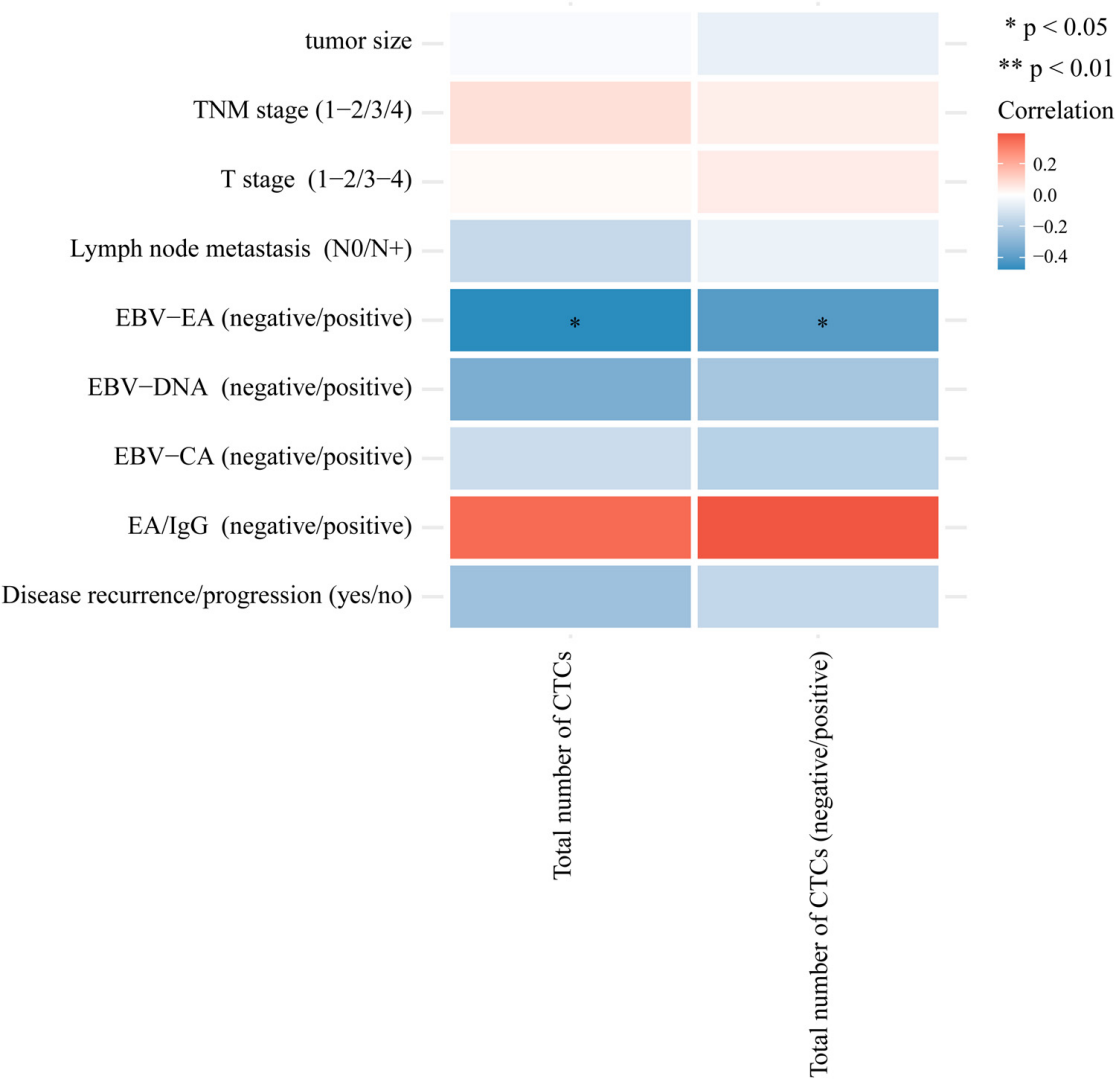
Relationship between CTCs and different clinical characteristics in patients undergoing radiotherapy. Red indicates positive correlation and blue indicates negative correlation.

Because MMP2 plays an important role in the EMT state transition of CTCs, we conducted 62 CTC sample tests to explore the relationship between MMP2 protein expression in CTCs and various clinical indicators of NPC, mainly analyzing the relationship between the number of MMP2 + CTCs, the number of MMP2 + mesenchymal CTCs, and the clinical characteristics previously described. Compared to before radiotherapy, the positive rate of MMP2 + total CTCs in patients with NPC increased after receiving radiotherapy. However, the positive rate of MMP2 + mesenchymal CTCs decreased during radiotherapy and then rose back to pre-radiotherapy levels after the treatment was completed (Table 5). After induction chemotherapy, the MMP2 protein levels on CTCs before and after radiotherapy were unrelated to clinical pathological indicators. In patients undergoing radiotherapy, the positivity of EBV-EA showed a negative correlation with MMP2 protein expression on CTCs, indicating that patients who are positive for EBV-EA tend to have a lower MMP2 protein expression detected on their CTCs (Figure 2).

**Table 5:** Relationship between MMP2 expression on CTCs and different clinical characteristics

Spearman Rho			MMP2+	
			Total number of CTCs	Total number of CTCs (negative/positive)
Tumor size	Correlation coefficient	*N* = 27	−0.018	−0.058
	*P* value		0.927	0.774
T stage (1–2/3–4)	Correlation coefficient	*N* = 27	0.015	0.045
	*P* value		0.941	0.825
Lymph node metastasis (N0/N+)	Correlation coefficient	*N* = 27	−0.150	−0.053
	*P* value		0.456	0.792
TNM stage (1–2/3/4)	Correlation coefficient	*N* = 27	0.075	0.039
	*P* value		0.711	0.845
EBV-CA (negative/positive)	Correlation coefficient	*N* = 26	−0.135	−0.185
	*P* value		0.511	0.365
EBV-EA (negative/positive)	Correlation coefficient	*N* = 26	−0.477	−0.409
	*P* value		0.022	0.038
EA/IgG (negative/positive)	Correlation coefficient	*N* = 19	0.349	0.391
	*P* value		0.144	0.098
EBV-DNA (negative/positive)	Correlation coefficient	*N* = 26	−0.329	−0.228
	*P* value		0.100	0.262
Disease recurrence/progression (yes/no)	Correlation coefficient	*N* = 26	−0.249	−0.158
	*P* value		0.220	0.440

### 3.4 Relationship between MMP2 protein expression on CTCs and their EMT status

**Figure 3:**
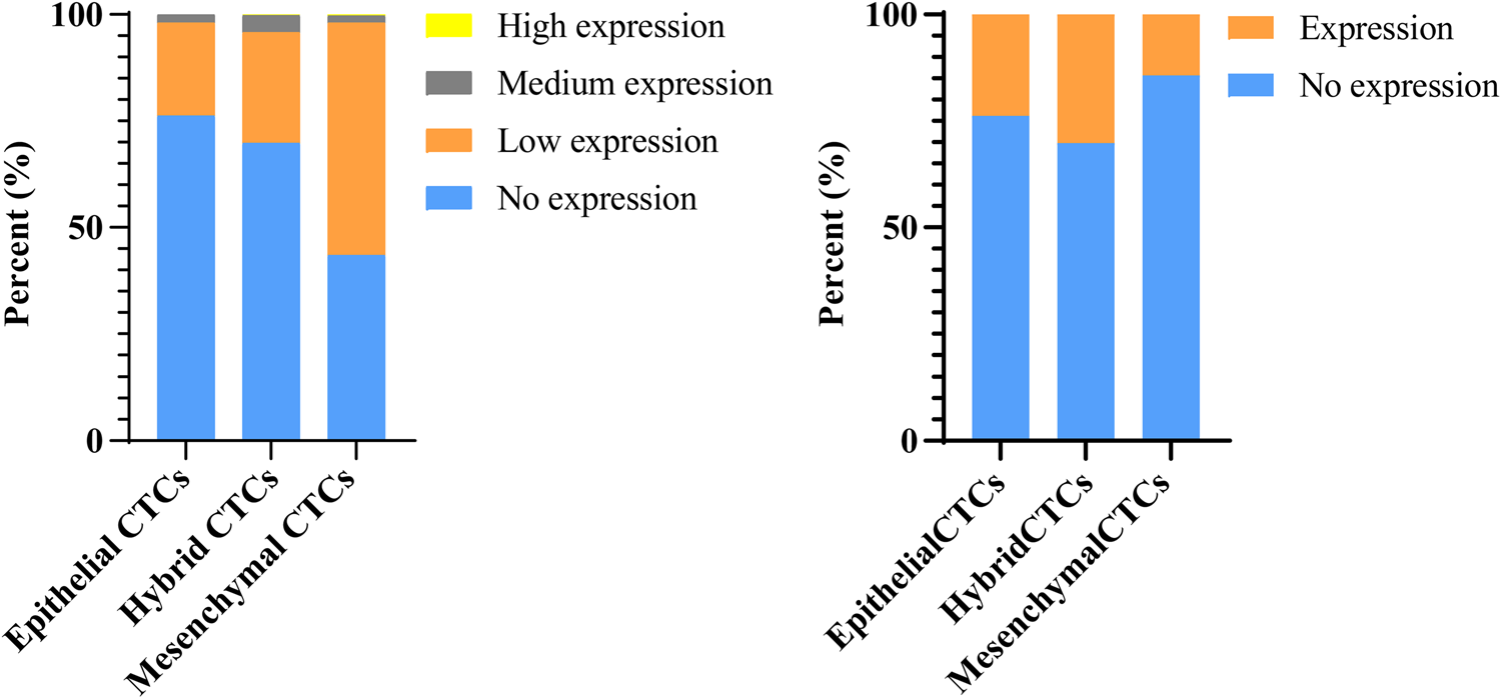
Comparison of MMP2 protein expression in different types of CTCs. The graph shows the distribution of epithelial-type, mesenchymal-type, and hybrid-type CTCs expressing MMP2 protein.

**Figure 4:**
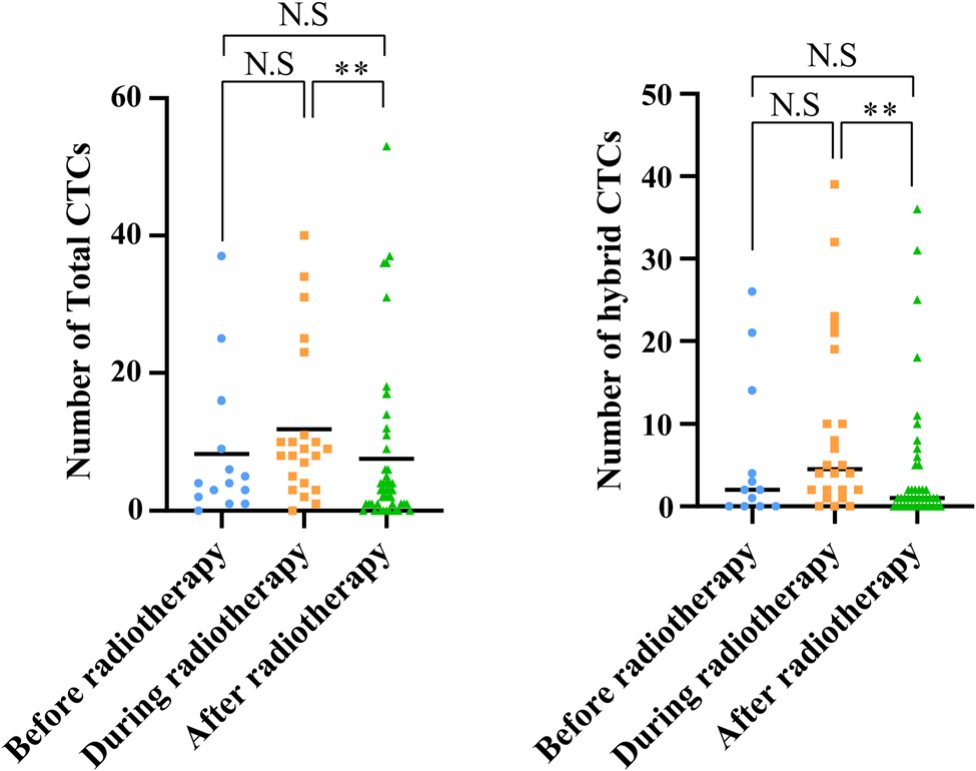
Changes in the total number of CTCs and the number of hybrid CTCs before, during, and after radiotherapy.

Among all the CTC test results, there were 24 cases of epithelial-type CTCs expressing MMP2 protein and 77 cases without MMP2 expression; 132 cases of mesenchymal-type CTCs expressing MMP2 and 22 cases without MMP2 expression; and 296 cases of hybrid-type CTCs expressing MMP2 and 128 cases without MMP2 expression. Chi-square test indicated that the positive protein expression rate of MMP2 in hybrid-type and epithelial-type CTCs was higher than that in mesenchymal-type CTCs, and the difference was statistically significant (*P* < 0.001, Figure 3). Moreover, comparing patients before, during, and after radiotherapy, it was found that the total number of CTCs and the number of hybrid-type CTCs increased during radiotherapy and decreased after the therapy, with the difference being statistically significant. The changes in the number of epithelial-type CTCs, mesenchymal-type CTCs, and MMP2 positive CTCs showed no significant differences (Figure 4).

## 4 Discussion

Recent progress in grasping the risk elements and mechanisms behind nasopharyngeal cancer, coupled with innovations in diagnostic and therapeutic methods, has created fresh avenues for the early identification and betterment of patient prognoses. The technique of liquid biopsy, which analyzes CTCs or cell-free DNA, emerges as a more economical and less intrusive method for the early detection of cancer, monitoring the effectiveness of treatments, and recognizing signs of tumor recurrence, when contrasted with traditional diagnostic procedures [19,20]. The clinical application of counting CTC has been extensively explored across a range of solid tumors, such as those found in the lung, prostate, breast, colorectal, head and neck, and pancreas [21–25]. Elevated count CTCs have been consistently linked with adverse outcomes, such as metastasis, treatment resistance, or recurrence of cancer, across multiple cancer types. For instance, in a study focusing on small-cell lung cancer, elevated CTC levels (≥10 CTCs per 5 mL of blood) were strongly linked to a more advanced TNM stage, including extensive lymph node and distant metastases, suggesting a poorer prognosis [26]. In research on hormone receptor-positive (HR+) metastatic breast cancer, findings indicated that patients with ≥5 CTCs per 7.5 mL of blood post-treatment experienced significantly poorer OS and progression-free survival (PFS) compared to those with fewer than five CTCs [27]. In NPC, baseline and post-treatment CTC levels, along with their longitudinal changes, were significantly linked to shorter PFS as determined by Kaplan–Meier analysis [28]. This study also found that nasopharyngeal cancer patients receiving radiotherapy with higher total counts of CTCs were more prone to relapse. Our results are consistent with previous studies and also demonstrate that high CTC counts are associated with nasopharyngeal cancer recurrence.

EMT involves the transformation of epithelial tumor cells, leading them to shed their cell-to-cell adhesion and develop mesenchymal characteristics that enhance invasiveness. In the course of spreading, tumor cells activate EMT to separate from the basement membrane and directly infiltrate the bloodstream [29–32]. The heterogeneity of CTCs reflects critical features related to metastatic progression, particularly EMT plasticity. During the EMT process, the phenotypes of CTCs transition dynamically between epithelial (E-CTCs), mesenchymal (M-CTCs), and hybrid forms (E/M-CTCs) [33,34]. In early-stage cancer, CTCs often mirror the primary tumor’s epithelial characteristics. Yet, as cancer progresses to advanced stages, the CTC profile shifts toward a mesenchymal phenotype due to the metastatic processes driven by EMT. This phenotypic flexibility, rooted in EMT, allows CTCs to navigate through various stages of metastasis, enhancing their drug resistance, survival, mobility, and invasive capabilities. Different states of EMT have been reported to contribute to cancer progression in different cancers [35–37]. For instance, a study indicates that a high total count of CTCs is associated with a poorer prognosis in all subtypes of lung cancer, and this effect intensifies as the detection threshold for CTCs increases. Moreover, epithelial CTCs have a greater prognostic value in lung cancer than CTCs of other EMT states [38]. Additional research indicates that in squamous cell carcinoma, the loss of FAT1 function facilitates tumor initiation, progression, invasiveness, stemness, and metastasis by triggering a hybrid state of EMT [39]. Counts of M-CTC and E/M-CTC are linked to the prognosis of patients with malignancies of the urinary system. Elevated numbers of M-CTC and E/M-CTC serve as indicators of a poorer prognosis in individuals suffering from urinary system [40]. In NPC research, a study demonstrated that 3 months after concurrent chemoradiotherapy, the total count of CTCs and epithelial/mesenchymal hybrid CTCs significantly decreased, while the counts of purely epithelial or purely mesenchymal CTCs did not decrease. Cases with an E/M hybrid dominance showed lower disease-free survival rates and distant metastasis-free survival rates compared to cases without E/M hybrid dominance [41]. In addition, previous studies also demonstrate that in NPC patients, higher counts of CTCs and mesenchymal CTCs are significantly correlated with advanced TNM staging, shorter PFS, and OS, making them valuable biomarkers for predicting patient outcomes and therapy response [42–44]. However, the changes in CTCs of different EMT statuses during radiotherapy following induction chemotherapy and their impact on prognosis remain unclear. This study suggested that during radiotherapy, the total count of CTCs and the number of hybrid CTCs increased, but these values decreased after the completion of radiotherapy, a difference that was statistically significant. In contrast, the changes in the number of epithelial CTCs and mesenchymal CTCs were not significant. Moreover, the total count of CTCs and the number of hybrid CTCs after radiotherapy were associated with patient disease progression or relapse. These results indicate that hybrid CTCs play a significant role in predicting the effectiveness of different treatment regimens and the prognosis of patients with NPC.

MMPs are enzymes capable of degrading components of the ECM, providing a physical pathway for tumor cell migration and invasion. During the EMT process, the expression of MMPs by tumor cells increases, aiding the cells in crossing the basement membrane and surrounding tissues, thereby promoting tumor invasion and metastasis [45,46]. In contrast to numerous studies on EMT in CTCs, there are few studies on MMPs expression in CTCs. In a study on breast cancer, MMP9 expression showed no significant difference across different EMT states, and there was no difference in prognosis between patients with varying EMT states of CTCs expressing different levels of MMP9 [47]. In this study, the results indicated that the positive expression of MMP2 on hybrid and epithelial CTCs was higher than on mesenchymal CTCs. However, after undergoing radiotherapy, the proportion of MMP2 + CTCs increased compared to before receiving radiotherapy, yet the count of MMP2 + CTCs was not associated with the prognosis of nasopharyngeal cancer patients. This result suggests that MMP2 may play a significant role in maintaining the epithelial state of EMT, or at least a partial epithelial characteristic, in nasopharyngeal cancer CTCs. However, the specific mechanisms behind this remain to be further investigated by us. Moreover, this study showed that the positivity rate of MMP2 in CTCs was not related to the recurrence of nasopharyngeal cancer in patients, a finding that could be attributed to the insufficient sample size of this study. Future studies will require larger cohorts to establish the relationship between MMP2 expression in CTCs and the prognosis of patients with NPC.

In conclusion, this study is the first to explore the relationship between different EMT states of CTCs and prognosis of nasopharyngeal cancer patients after induction chemotherapy followed by radiotherapy. In this context, the count of hybrid CTCs and the expression of MMP2 in these types of CTCs may play a significant role, which could be of great importance for guiding the treatment and monitoring disease progression in nasopharyngeal cancer.
